# Rating Traits Together or Apart: Presentation Format Affects First Impression Judgements

**DOI:** 10.1177/17470218261417512

**Published:** 2026-01-12

**Authors:** Nadine Lavan, Jessica H. Turner, Matthew R. Utber, Clare A. M. Sutherland

**Affiliations:** 1 Department of Biological and Experimental Psychology, School of Biological and Behavioural Sciences, Queen Mary University of London, UK; 2 School of Psychology, University of Aberdeen, King’s College, Scotland, UK; 3 School of Psychological Science, University of Western Australia, Australia

**Keywords:** first impressions, perceptual ratings, task design, face perception

## Abstract

In perception experiments, researchers often collect multiple perceptual judgements from the same stimuli and participants to answer their research questions. While the way in which perceptual judgements are collected may affect the data, research rarely considers task design when investigating this topic. We therefore investigated how two frequently used methods of collecting multiple ratings can affect perceptual judgements, focusing on first impressions of faces. One participant group provided ratings of seven person characteristics (e.g. femininity, youthfulness, trustworthiness) blocked by characteristic, rating faces for one person characteristic at a time, across seven blocks. Another participant group completed one block in which they rated all seven characteristics simultaneously via a list of rating scales. The listed presentation format reduced task duration by 22%, but affected the perceptual ratings in several ways, pointing to reduced data quality, potentially as a result of satisficing behaviours. Specically, inter-rater agreement was lower for some person characteristics (youthfulness, femininity, and trustworthiness) in the listed format. Variance in ratings was also reduced for youthfulness, femininity, and dominance, with ratings clustering closer to the middle of the scale. Importantly, correlations between different person characteristics became universally positive in the listed format, indicating reduced independence of judgements. These findings highlight that while presenting multiple judgements at the same time may offer efficiency, this approach can introduce systematic biases and potentially reduce the reliability of perceptual data. We therefore suggest using a blocked presentation format and consider how these trade-offs would impact experiments looking at multiple perceptual judgements collected from the same participants.

## Introduction

In many experimental and social psychology experiments, researchers collect perceptual judgements from stimuli via ratings from participants. In many of these studies, the stimuli (e.g. auditory or visual stimuli) are assessed on multiple different perceptual characteristics. For example, researchers interested in how participants form first impressions may collect ratings of dominance, trustworthiness, competence, and more for their chosen stimuli from participants (Hehman et al., 2017; Lavan, 2023; Lavan & Sutherland, 2024; McAleer et al., 2014; Mileva et al., 2019; Oh et al., 2020, 2022; Oosterhof & Todorov, 2008; Stolier, Hehman, Keller, et al., 2018; Sutherland et al., 2013, 2018; Tsantani et al., 2023). Researchers interested in emotion perception will often collect multiple ratings for emotion intensity and other affective measures, such as arousal, valence, and authenticity, from the same stimuli (Lima et al., 2013, 2014; Pinheiro et al., 2021; Riediger et al., 2011; Sauter et al., 2010). Similarly, researchers interested in word processing and learning will often assess their word stimuli based on measures such as concreteness, imaginability, and context availability (Bradley & Lang, 1999; Yao et al., 2017; Yee, 2017).

Many experiments collect perceptual judgements or ratings in such a way that a participant sees or hears a stimulus and then provides a judgement for a single perceptual characteristic (e.g. seeing a face and then providing a judgement of how old that face looks). When a study requires judgements for multiple perceptual characteristics, researchers typically adopt one of two approaches: a between-subjects design, where researchers recruit separate groups of participants, each assigned to provide a different perceptual characteristic (Collova et al., 2019; Oosterhof & Todorov, 2008; Sutherland et al., 2018). Alternatively, in a within-subjects design, the same participants are often asked to evaluate multiple characteristics by presenting the same stimulus multiple times in separate blocks (Lavan, 2023; Lavan et al., 2021; Lin et al., 2021; Mileva & Lavan, 2023; Sutherland et al., 2020). In the latter case, each block focuses on a distinct perceptual judgement – participants might first rate a set of faces for perceived age and later, in a different block, rate the same face for perceived competence (for instance). The reasoning behind only collecting one judgement per stimulus presentation often revolves around the desire to maintain a level of independence for the measurements of the different judgements: In a between-subjects design, participants only ever judge a stimulus once for one characteristic, such that ratings of the other characteristics are by definition independent between the different groups of participants. The best approximation of the independence of judgements in the context of a within-subjects design can be achieved by separating multiple judgements in times, where a judgement of how old a specific person looks is not likely to substantially influence the judgement of how competent that particular person looks, if these two judgements are separated by the participant rating a number of other faces for age, competence, or other characteristics.

Whether a between- or within-subjects approach is chosen often depends on the specific research question asked: For example, if researchers are interested in describing the characteristics of a set of stimuli, when validating stimuli for a database, when collecting large-scale data, or when trying to build a general model of first impression formation that minimised the role of participant-specific effects, a between-subjects design might be sufficient or even the preferred design. A within-subjects design is preferable if researchers are interested in how the (individual) participants perceive multiple characteristics in relation to one another or when investigating individual differences.

In studies where researchers gather multiple perceptual judgements about a set of stimuli, there is often a desire to increase the efficiency of the experimental design by streamlining tasks to collect a maximum amount of data in the least time possible. One alternative approach is to present each stimulus only once and collect several perceptual judgements during that single exposure. This method is intuitively more efficient as it reduces the number of stimulus presentations, which should shorten the overall duration of the experiment. Shorter experiments can then, in turn, help reduce participant fatigue and boredom.

This approach can also be particularly advantageous studies that rely on stimuli being experienced by participants for the first time or only a single time: For example, in studies of first impressions or studies requiring participants to be entirely unfamiliar with the stimuli, limiting stimulus exposure to a single presentation can be theoretically important and may also help preserve the integrity of the participants’ initial impressions. As a result, perhaps, this approach has been frequently used in early first impression research, in the form of semantic-differential scales, where participants assess a person or group of people on a number of bipolar scales (e.g. cruel – kind; foolish – wise; Kervyn et al., 2013; Osgood, 1964; Stoklasa et al., 2019). However, this efficiency comes at the same time with potential disadvantages. When multiple judgements are made based on a single presentation of a stimulus, these judgements may not be truly independent. In line with the argument in favour of collecting only one perceptual judgement per stimulus presentation, making one judgement could influence subsequent judgements, leading to interdependence among responses. This interdependence could artificially inflate observed relationships between variables or could even introduce spurious correlations created by the experimental task design. Similarly, increased interdependence might exacerbate order effects (Tourangeau et al., 2004). For example, if participants give a high rating of attractiveness to a face, they may also opt for high trustworthiness and competence ratings. This would be in line with halo and overgeneralisation effects, where the perception of one (positive) characteristic often influences the perception of other characteristics and has led to rich insights into perception (Forgas & Laham, 2016; Montepare & Dobish, 2003; Nisbett & Wilson, 1977; Zebrowitz & Montepare, 2008).

This example of potential halo and overgeneralisation effects shows that this concern is not merely methodological, but has important implications for theory development, heightened by the fact that many person characteristics are themselves inherently correlated in human perception. Indeed, the interdependence of person characteristics during first impression formation is a problem (or feature) that has stimulated many theoretically influential approaches in first impressions research on faces, including the building of lower-dimensional models and higher-dimensional matrix investigations of impressions (Lavan, 2023; Oh et al., 2022; Stolier, Hehman, Keller, et al., 2018). Similarly, the interdependence of first impressions has sparked investigations into the holistic nature of impression formation (Vernon et al., 2014) and into the accuracy of first impressions (Foo et al., 2022; Skuk et al., 2024).

Numerous studies have tackled the question of how different aspects of the nature of rating scales and other response options affect perceptual judgements (Kusmaryono et al., 2022; South et al., 2022; Wakita et al., 2012). Similarly, there is a substantial literature on questionnaire design and validation that examines how grouping versus randomising questionnaire items of a given construct (e.g. items measuring one of the Big 5 traits in a personality questionnaire) affects data. Studies here have found that when items measuring the same concept are grouped, these items become more correlated, while randomisation can reduce inter-item correlations (Kusmaryono et al., 2022; Tourangeau et al., 2004; Weijters et al., 2010, 2014). To our knowledge, no empirical work in psychology has systematically assessed whether and how collecting a single versus multiple perceptual judgements per stimulus presentation affects these judgements in a within-subjects design. Yet, design choices may affect our data and thus the conclusions we can draw from them.

In our experiment, we therefore set out to assess how much these experimental design choices affect perceptual judgements, using first impression formation from faces as a test case. We used first impressions due to the theoretically important nature of understanding intercorrelations between different impressions from faces. For this purpose, we collected femininity, youthfulness, warmth, competence, attractiveness, dominance, and trustworthiness ratings from participants on 9-point Likert scales. These seven characteristics were chosen as they have been identified as one of the underlying dimensions of low-dimensional trait spaces (Lin et al., 2021; Oosterhof & Todorov, 2008; Sutherland et al., 2013). One group of participants completed these ratings in a blocked presentation format, where they were presented with a face, which they rated for one of the seven person characteristics above. This block was followed by six further blocks to obtain ratings from all the different characteristics. This presentation format thus maximised the independence of judgements across characteristics. The other group of participants completed the ratings in a listed presentation format, where they were presented with a face once and completed all seven ratings via a list of Likert scales for all seven person characteristics. This presentation format thus maximises efficiency by reducing the stimulus presentations from seven (see the blocked design) to a single presentation.

We predicted that while the experiment duration would be shorter for the listed than for the blocked presentation format, the presentation format would affect the first impression judgements we collected. Specifically, we expected that the first impressions of the different person characteristics would influence each other more in the listed than in the blocked condition, that is, become more intercorrelated (Tourangeau et al., 2004; Weijters et al., 2010, 2014). As a general aim of the study, we intended to better understand what, if any, differences arose between the two presentation formats, without having directional predictions. To do this, we compared inter-rater agreement to assess the overall data quality, mean ratings per stimulus and the associated variance between groups, and correlated mean ratings per characteristic across presentation format as well as within presentation format, across characteristics.

## Methods

### Participants

Sixty participants took part in this experiment. All participants were recruited from Prolific.co, were aged between 18 and 65 years old, and were all native speakers of English. All participants were paid at a rate of £10 per hr. The experiment was approved by the local ethics committee at Queen Mary University of London. Twenty-eight of these participants (mean age = 37.7 years, *SD* = 12.9 years, 16 female) were assigned to the blocked condition, 32 participants (mean age = 30.0 years; *SD* = 5.4 years, 15 female) completed the ratings in one block, via the listed condition (see “Procedure” for details). These sample sizes were chosen to be able to capture a relatively stable mean rating per item (Hehman et al., 2025).

We did not need to exclude any participants based on the vigilance trials (see “Procedure”). We also assessed whether participants were given variable responses, where we excluded the ratings of person characteristics, where participants gave the same response for over 80% of trials. In the blocked conditions, we excluded data from one participant for competence ratings. In the listed condition, we excluded data for competence ratings from one participant, youthfulness ratings from another, and competence, dominance, and trustworthiness ratings from a third participant. Final sample sizes per characteristic thus ranged from 26 ratings (for competence after 2 exclusions), and 28 ratings for the characteristics where no exclusions were made.

### Materials

Participants rated 60 face stimuli in this task (30 female faces). The face stimuli were selected from the 10k U.S. Adult Faces Database (Bainbridge et al., 2013), a well-used database of naturalistic face images taken from the internet. Images are shown with an oval mask and on a white background. All images were selected such that faces that were looking at the camera were front-facing and had good image quality. To facilitate a wide range of first impressions, there was otherwise no constraint on the ethnicity, age, expressed emotion, or any other characteristics of the face images according to the background measures provided for the database used. The final 60 images were selected based on being listed as the first 30 male and female faces, respectively, in the database that fit the selection criteria above. The faces were unfamiliar to participants (confirmed via a debrief question).

### Task and Procedure

The experiment was run using Gorilla Experiment Builder (Anwyl-Irvine et al., 2020). Participants first read an information sheet and then provided informed consent. All participants then started the ratings task during which they rated the 60 face images for 7 different person characteristics (femininity, youthfulness, competence, warmth, attractiveness, dominance, and trustworthiness). These seven characteristics were chosen in line with influential models of first impressions from adult faces. Specifically, the first four person characteristics were selected from Lin et al. (2021) as four dimensions defining face trait space, while attractiveness, dominance, and trustworthiness are partially overlapping person characteristics that have frequently been used in other studies of face perception (Oosterhof & Todorov, 2008; Sutherland et al., 2013). Competence, warmth, attractiveness, dominance, and trustworthiness scales ranged from 1 – “*Not at all [characteristic]*” to 9 – “*Very [characteristic]*.” Youthfulness ranged from 1 – “*young adult*” to 9 – “*old adult*,” femininity ranged from 1 – “*very masculine*” to 9 – “*very feminine*.”

**Figure 1.**
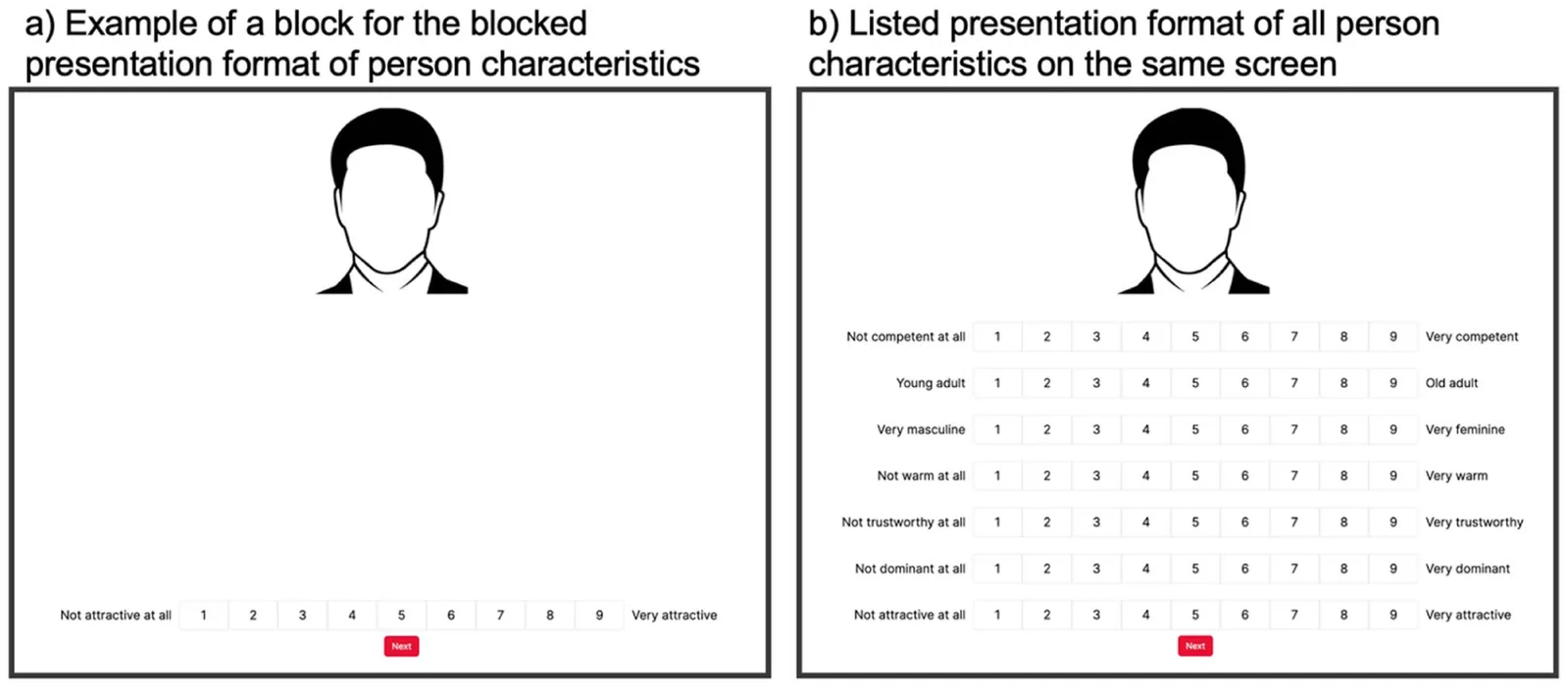
Illustration of the ratings task in the blocked presentation (panel a) and the listed presentation (panel b): (a) Example of a block for the blocked presentation format of person characteristics, (b) Listed presentation format of all person characteristics on the same screen.

Participants were then randomly assigned to one of the two versions of the ratings task:

(1) A blocked presentation format, where participants were presented with and rated all 60 faces for a single person characteristic in a block (Figure 1a). To rate all person characteristics in this version of the task, participants thus completed seven blocks (with the same faces repeated in each block). The order in which the faces were presented was fully randomised, as was the order of blocks.
(2) A listed presentation format, where participants were presented with and rated all 60 faces for all seven person characteristics at once, presented as a list of rating scales, on the same screen (Figure 1b). To rate all person characteristics in this version of the task, participants thus completed one block. The order in which the faces were presented was also fully randomised. To control for order effects and reduce the potential for “anchoring,” we furthermore created 10 lists that have different orders of person characteristics. In these 10 orders, we ensured that all person characteristics were presented in each of the 7 positions 1–2 times. We also ensured that specific pairs of identities in adjacent positions (e.g. “attractiveness” followed by “youthfulness” in the list of ratings) did not occur more than 3 times.

For both versions of the tasks, faces were visible alongside the rating scale(s) until participants moved on to the next trial to minimise differences in working memory demands. Responses were self-timed. There were 6 vigilance trials per block: During vigilance trials, instead of being shown an image of a face to rate, participants were shown text on the screen, instructing them to provide a specific rating (e.g. “Please select 9”).

### Analysis Plan

We first examined the total time taken for participants to complete all ratings within the experiment to establish whether and to what degree it was truly more efficient to present different characteristics in a list.

We examined inter-rater agreement, since experiments collecting first impressions via rating scales view high inter-rater agreement as a marker of good data quality for studies interested in impression formation at a group level, given that aspects of first impressions are to a degree shared between participants (Oosterhof & Todorov, 2008; Sutherland et al., 2018, but see Hehman et al., 2017; Lavan & Sutherland, 2024; Martinez et al., 2020; Sutherland et al., 2020). To this end, we therefore compared inter-rater agreement for each characteristic by computing the ICC(2,k).

We then looked at mean ratings and the associated variance across different stimuli, as the field has often been interested in comparing mean impression ratings of perceptual judgements (e.g. across participant groups or stimulus sets; Bjornsdottir & Beacon, 2024; Tsantani et al., 2023; Zebrowitz et al., 1993). Similarly, studies have often looked to understand and model the (co)variance of impression formation (Hehman et al., 2019; Lavan et al., 2024; Oh et al., 2019, 2020; Stolier, Hehman, & Freeman, 2018; Stolier, Hehman, Keller, et al., 2018; Sutherland et al., 2016; Walker & Vetter, 2016), where appropriately capturing variation in perceptual judgements across different faces is also a key marker of good data quality. Here, we first compared mean ratings using *t*-tests and the associated variance using Levene’s tests across all stimuli between presentation formats. We followed this up with correlations between mean ratings per item for each characteristic to assess how much the mean ratings are affected by presentation format, when considering individual items.

Finally, we assessed how the relationship among ratings for the different characteristics may be affected by the different presentation formats, given that the blocked presentation format affords more independence to ratings, while the listed presentation format may be prone to introducing co-dependencies between characteristics. We computed pairwise Spearman’s correlation between all pairs of characteristics to create representational (dis)similarity matrices for the face ratings from the blocked and listed presentation conditions, respectively. This allows us to conclude to what degree and in what way first impression judgements are interdependent on one another.

## Results

### Participants Complete the Experiment Faster When Being Presented With Listed Characteristics

One motivation to present multiple ratings in a listed presentation format is the appeal of participants potentially completing the experiment more quickly, given the arguably more streamlined design due to fewer presentations of the stimuli and fewer instruction screens.

In our experiment, we measured the time taken from the very first rating to the last rating. Participants took 26:06 min on average to complete all ratings in the listed presentation condition and took 33:07 min on average in the individual presentation condition. Participants in the listed condition, therefore, completed the experiment in 22% less time than participants in the blocked condition (*t*(58) = 3.08, *p* = .003), suggesting increased efficiency of this presentation format.

### Inter-Rater Agreement is Lower for Some Characteristics in the Listed Presentation Format

In our experiment, inter-rater agreement was overall good (ICC[2,k] > .75; Koo & Li, 2016) for many of the investigated person characteristics, particularly in the blocked presentation. For a minority of person characteristics, inter-rater agreement was moderate (ICC[2,k] = .5 − .75) or low (ICC[2,k] < .5; see Table 1 for details).

**Table 1.** ICC(2,k)s and 95% Confidence Intervals for the Ratings of the Person Characteristics by Condition.

	Blocked presentation		Listed presentation	
Person Characteristic	ICC(2,k)	95% CIs	ICC(2,k)	95% CIs
Warmth	0.90	[0.80, 0.93]	0.86	[0.80, 0.91]
Competence	0.55	[0.38, 0.69]	0.45	[0.25, 0.62]
Youthfulness	0.97	[0.96, 0.98]	0.69	[0.57, 0.79]
Femininity	0.99	[0.99, 0.99]	0.86	[0.81, 0.91]
Attractiveness	0.80	[0.71, 0.87]	0.78	[0.70, 0.86]
Dominance	0.80	[0.72, 0.87]	0.71	[0.60, 0.80]
Trustworthiness	0.76	[0.66, 0.84]	0.52	[0.34, 0.68]

We directly compared agreement for the two presentation formats: If 95% confidence intervals (CIs) around the ICCs did not overlap, we conservatively inferred that inter-rater agreement differed between presentation formats. While there is no clear one-to-one mapping from CIs to *p*-values, CIs that touch but do not overlap are comparable to an α level of *p* = .01. If desired, this “rule of thumb” may be used to roughly align our findings with *p*-value-based inferences (Cumming, 2014; Lavan & Sutherland, 2024; Tan & Tan, 2010).

95% CIs did not overlap for youthfulness and femininity 95% confidence intervals, with the ICCs being substantially lower for the listed presentation than for the blocked presentation (see Table 1). For warmth, competence, attractiveness, trustworthiness, and dominance, 95% CIs for the ICCs overlapped. While inter-rater agreement was less than 0.1 lower for warmth, attractiveness, and dominance for the listed presentation, inter-rater agreement differed markedly for trustworthiness (ICC[2,k] = .76 for the blocked presentation and .52 for the listed presentation). Overall, we therefore conclude that inter-rater agreement was somewhat lower for the listed presentation, with the size of these differences varying between person characteristics.

### Variability in Ratings is Reduced in the Listed Presentation Format, While Group Means are Unaffected

To assess how presentation format (blocked vs. listed) affects mean ratings per face image and their distributions, we ran independent samples Welch *t*-tests to compare mean ratings per stimulus and Levene’s tests to test for homogeneity of variances between the two presentation formats. We ran these tests for each person characteristic, such that alpha was Bonferroni-adjusted for 7 comparisons (alpha = .007).

**Figure 2.**
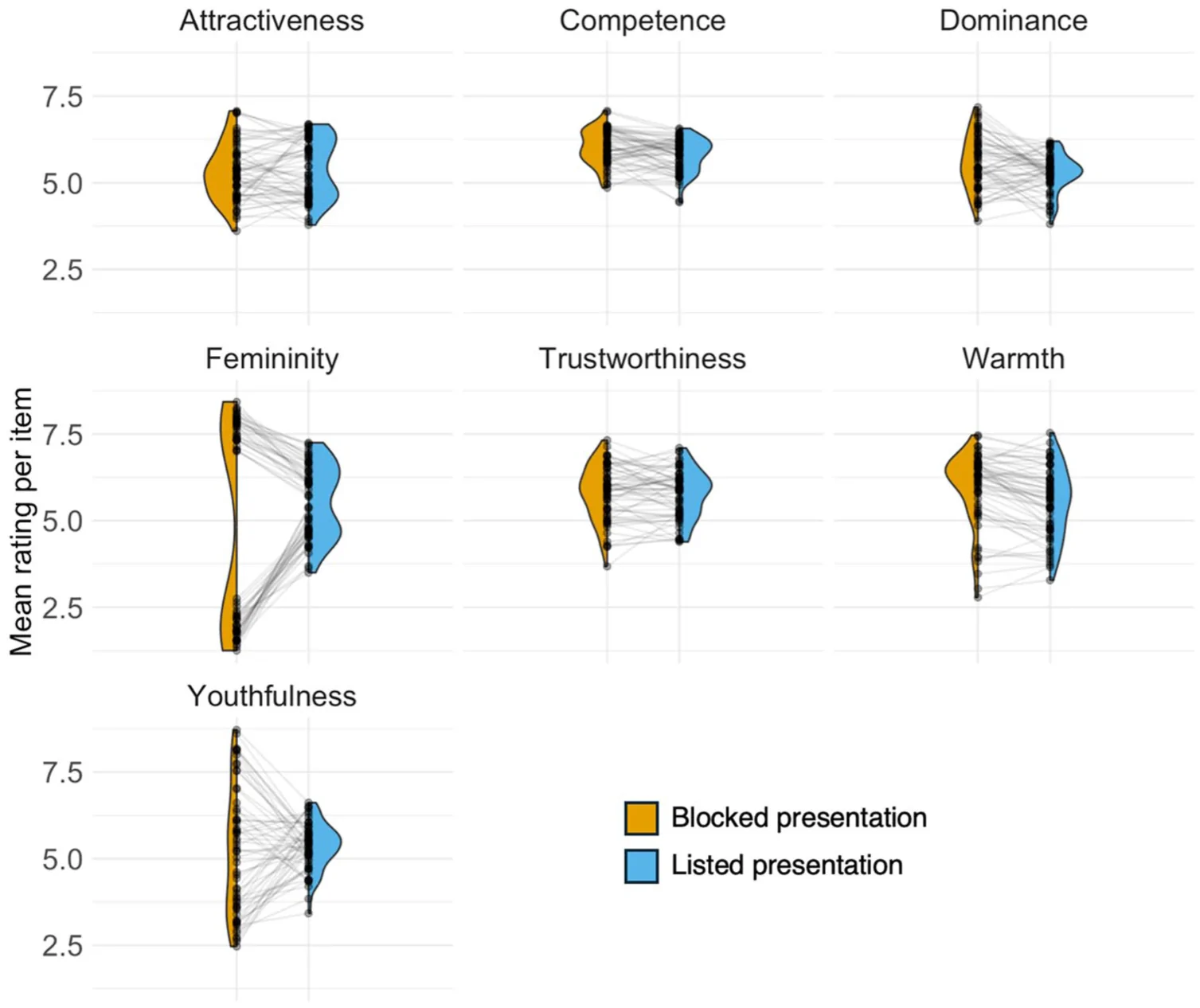
Plots of mean ratings per item by presentation format for all characteristics. Black dots show mean ratings per item, half-violin in blue and yellow show the distribution of data points, and grey lines connect the data points for the same items to illustrate any differences in mean ratings introduced by presentation format.

None of the *t*-tests were significant (all *p*s > .01, see Table 2 for details), suggesting that overall mean ratings were unaffected by presentation format. As for the inter-rater agreement, the variance of ratings for some characteristics was affected by presentation format (Figure 2). Levene’s test, however, showed heterogeneity of variances for femininity, youthfulness, and dominance (*F*s[1,118] > 8.4, *p*s < .005), with the variance for the listed presentation being lower than for the blocked presentation. Variances were similar across presentation formats for the remaining characteristics of attractiveness, warmth, trustworthiness, and competence (*F*s[1,118] < 1.90, *p*s > .170, see Table 2 for details).

**Table 2.** Levene’s Tests and *t*-Tests on Mean Ratings for Each Characteristic, Contrasting the Blocked and Listed Presentation Formats.

Person Characteristic	Levene’s *p*	*t* value	*p* value	Cohen’s *d*	Mean rating [*SD*] (listed)	Mean rating [*SD*] (blocked)
Femininity	<.001	−1.80	.076	−0.33	5.53 [1.04]	4.80 [2.94]
Youthfulness	<.001	−0.64	.524	−0.12	5.35 [0.68]	5.19 [1.82]
Attractiveness	.171	−0.63	.530	−0.11	5.41 [0.87]	5.31 [0.83]
Competence	.623	2.58	.011	0.47	5.78 [0.49]	6.02 [0.52]
Warmth	.545	2.19	.031	0.40	5.51 [1.02]	5.93 [1.07]
Trustworthiness	.385	0.25	.804	0.05	5.76 [0.70]	5.80 [0.81]
Dominance	.004	2.52	.013	0.46	5.28 [0.55]	5.59 [0.78]

### Presentation Format Differentially Affects Mean Ratings for Different Characteristics

**Figure 3.**
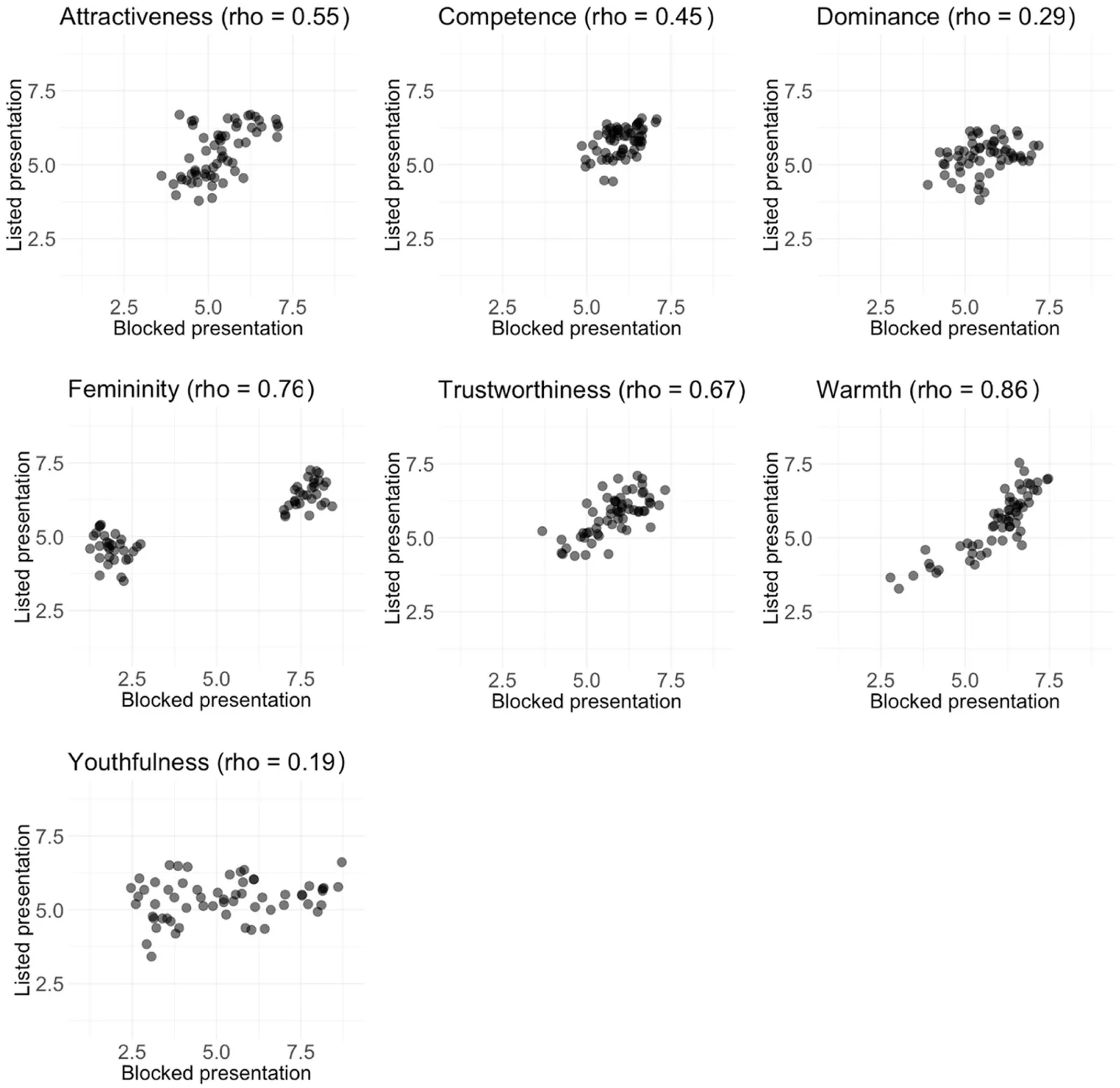
Scatterplots of mean ratings per face image for the blocked versus listed presentation format per characteristic.

Having found differences between the two presentation formats, we further compared them by running correlations between the mean ratings per face image for blocked versus listed presentation order for each characteristic (Figure 3). Given that participants rated the same face images for the same person characteristics, we would expect high correlations here if presentation format had no or only a little effect on the perceptual judgements. As in the previous analyses, different characteristics were affected differentially by the different presentation formats. There was no significant relationship for youthfulness ratings (rho[58] = .19, *p* = .147, CI [−0.07, 0.42]), and we furthermore observed only a weak, albeit significant correlation across presentation format for dominance ratings (rho[58] = .29, *p* = .024, CI [0.04, 0.51]). Correlations for dominance and competence were moderate (rhos[58] = .45, *p* = .003, CI [0.22, 0.63] and .55, *p* = .024, CI [0.34, 0.70], respectively). Warmth, femininity, and trustworthiness ratings were strongly correlated across presentation formats (warmth: rho[58] = .86, *p* < .001, CI [0.77, 0.91]; trustworthiness: rho[58] = 67, *p* < .001, CI [0.50, 0.79]; femininity = rho[58] = .76, *p* < .001, CI [0.63, 0.85]; see Figure 3 for details). Overall, mean perceptual ratings for faces were therefore related across presentation formats, expect for youthfulness, with correlation coefficients ranging from weak to strong, underlining the differential effect of presentation format on perceptual ratings.

### Correlations Between Characteristics are Inflated for Listed Presentation

**Figure 4.**
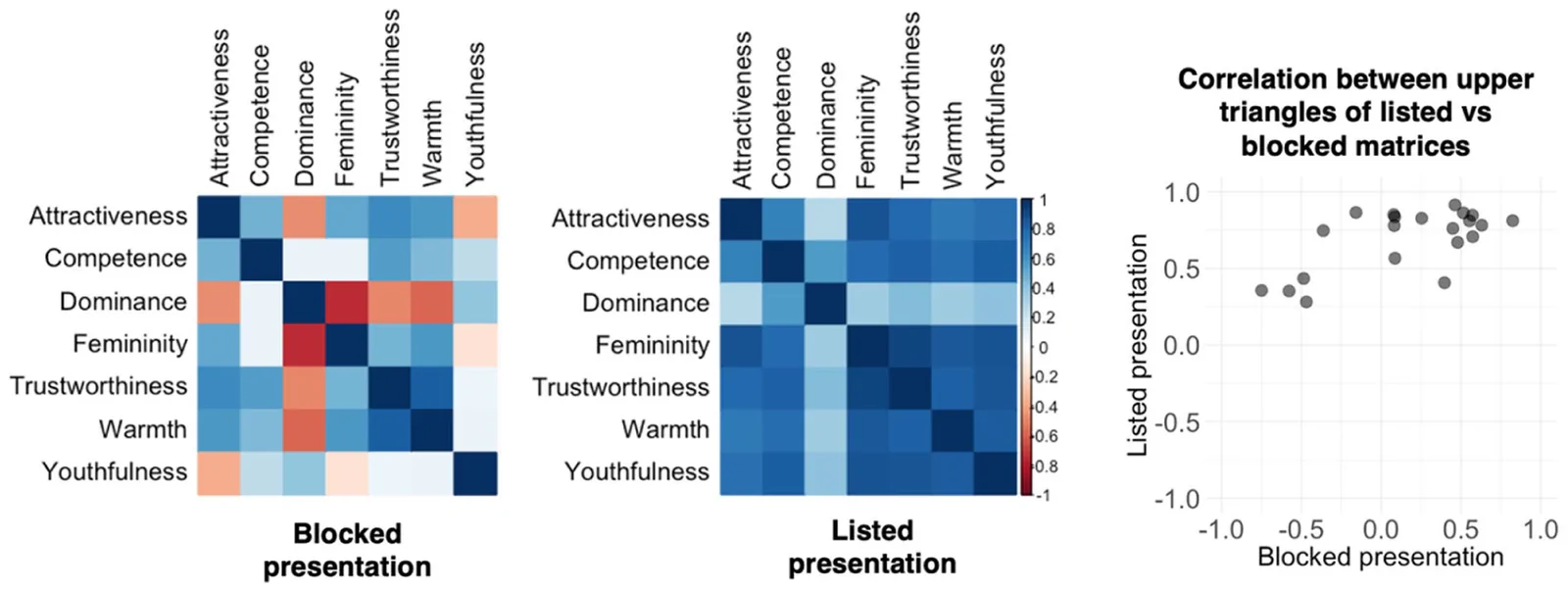
Representational (dis)similarity matrices for the face ratings collected via blocked and listed presentation formats. The scatterplot shows the correlation between the upper triangles of the two matrices.

To assess how the relationship among ratings for the different characteristics may be affected by the different presentation formats, we computed pairwise Spearman’s correlation between all pairs of characteristics to create representational (dis)similarity matrices for the face ratings from the blocked and listed presentation conditions, respectively (see Figure 4). These matrices are symmetrical, such that we only consider the (vectorised) correlation coefficients from the upper triangle, excluding the diagonal, in the analysis below.

We observe that correlation values are more varied (ranging from strong negative to strong positive correlations) for the blocked presentation (Levene’s test: *F*[1,40] = 11.33, *p* = .002). Similarly, on average, correlation coefficients between characteristics are lower for the blocked than for the listed presentation (*t*[27.42] = 4.90, *p* < .001, CI [0.31, 0.76]). This overall shift towards characteristics being more positively correlated then leads to striking changes in the overall structure of the matrix: For example, youthfulness and attractiveness are negatively correlated in the blocked presentation (i.e. with the youthfulness scale ranging from 1 – *young adult* to 9 – *old adult*, older people are rated as less attractive) but become strongly positively correlated in the listed presentation (i.e. older people are rated as more attractive). Similarly, dominance and femininity are negatively correlated in the blocked presentation (with the femininity scale ranging from 1 – *very masculine* to 9 – *very feminine*, more dominant faces were also perceived as less feminine), but become weakly positively correlated in the listed presentation condition (higher dominance associated with higher femininity). The negative correlations observed in the blocked condition are in alignment with a wealth of previous research (He et al., 2021; Korthase & Trenholme, 1982; Sutherland et al., 2015), whereas the positive correlations are not (to the best of our knowledge).

Despite these differences in how different pairs of characteristics are correlated with one another, there is, however, still some shared information present in the representational similarity matrices across presentation format, which we observe when correlating their respective upper triangles (rho = .42, *p* = .056, CI [0.00, 0.72]). While the correlation coefficient indicates a moderate relationship, the correlation is not statistically significant, likely due to a lack of power as a result of the limited number of data points in the correlation analysis.

## Discussion and Conclusion

We investigated how task design, specifically blocked or listed presentation format, affects perceptual ratings in a first impressions task and show that presentation format affects the experiment and data collected in various ways. We first found that participants completed the task 22% quicker in the listed presentation format. At the same time, however, inter-rater agreement was consistently lower for this condition, a trend that was more pronounced for some characteristics (e.g. trustworthiness and youthfulness) than others. These two overarching findings thus already highlight that presentation format impacted our data in complex ways. Below, we will discuss and evaluate our findings in detail.

When directly comparing mean ratings for each characteristic across the two presentation formats, we first find that these mean ratings largely remained the same. However, the variance associated with these mean ratings per face image was substantially reduced for the listed compared to the blocked presentation format, particularly in the context of ratings, where a substantial variance was expected (e.g. femininity and youthfulness impressions for a set of face images that included male and female faces of a wide age range). The differences in variances can be explained by mean ratings for individual face images cluster more closely around the group mean for some of the characteristics (femininity, youthfulness, dominance) in the listed presentation (see Figure 2). Given the subjectivity in many impressions (Foo et al., 2022), it is hard to make a strong claim about which measure of variation is more accurate; nevertheless, we can make some suggestions. Given that half of the images were selected to show male faces while the other half showed female faces, and given a substantial range of ages being present among the 60 faces, it is reasonable to expect a wide range of means for at least the femininity and youthfulness data when looking at data of good quality. A reduced range of mean ratings, therefore, suggests that this presentation style likely substantially influenced ratings, such that they converged towards the middle of the scale when averaging across participants. Reduced range in ratings where we could expect a wider range may thus indicate poorer quality data in the listed presentation condition. Furthermore, in the blocked condition, participants view the entire set of faces when rating the faces for the first person characteristic. This exposure provides a clear sense of the sample’s overall distribution and variance, which may influence ratings for subsequent characteristics. By contrast, in the listed condition, participants encounter faces sequentially without knowing what the remaining stimuli will look like. This could be another contributing factor, beyond poorer data quality, that may explain why there is less variance in mean ratings for the listed condition, reflecting uncertainty about whether future faces will differ substantially (e.g. in age or other characteristics).

We also find that correlations between the mean ratings per face image for the two presentation formats range from weak (and non-significant) to strong (and highly significant) across the seven person characteristics. If presentation format had not had a substantial effect on ratings, we would have expected to see that ratings for each characteristic in blocked and listed presentation formats would have been highly correlated. Finding this wide range of correlation strengths between mean ratings per face thus underlines one of the key findings from our study: Although presentation format substantially affects perceptual judgements for some person characteristics, others appear to be largely unaffected by presentation format (see warmth, which may be related to the notion of a primacy of warmth, Fiske et al., 2007). As a cautionary note, warmth appeared to be an exception in terms of not being affected by presentation format, such that perceptual judgements of most characteristics were affected in themselves and in relation to warmth and other characteristics. Thus, researchers will need to consider the relationships between traits as well as individual traits when deciding between a blocked or listed presentation format. Overall, it is, however, not easy to explain why certain characteristics were affected more than others: For example, given that femininity and youthfulness perception tend to be clearly linked to specific features of faces and are associated with relatively little idiosyncrasy in how they are evaluated (Lavan & Sutherland, 2024), we may have expected such “apparent” characteristics to be affected less than “inferred” characteristics, such as trustworthiness or competence, which are associated with more idiosyncratic judgements. Similarly, we might have expected dominance, alongside warmth, to be relatively stable, as a characteristic, given that it has been consistently proposed as a fundamental dimension of trait space (Lin et al., 2021; Mileva, 2025; Oosterhof & Todorov, 2008; Sutherland et al., 2013). This was, however, not the case with youthfulness and dominance being among the characteristics for which ratings changed most according to presentation style. We therefore cannot offer a conclusive cognitive or psychological explanation for why different characteristics were affected in different ways.

Beyond comparisons of how ratings for individual characteristics behaved for the two presentation formats, we also found that the relationships among person characteristics also differed depending on the presentation format. The blocked format replicated relationships between pairs of characteristics reported previously in the literature. For example, older people were perceived as less healthy, and less feminine faces were perceived as less dominant (Lavan, 2023; Lavan et al., 2024; Sutherland et al., 2013, 2015). For the listed presentation format, all relationships were shifted towards being positive relationships, such that the data suggest that participants considered older people to be healthier than younger people, and more feminine faces were perceived to be more dominant. While it is possible that halo or overgeneralisation effects (Forgas & Laham, 2016; Montepare & Dobish, 2003; Nisbett & Wilson, 1977; Zebrowitz et al., 1993) could underpin the universal shift toward positive correlations between characteristics in the listed format, we believe that it is more likely to reflect response-level processes rather than perceptual changes. For example, it is unlikely that well-documented relationships are unlikely to reverse simply due to presentation format. In fact, when characteristics are presented together and would become more interdependent, as would be the case for halo or overgeneralisation effects, we would have expected exaggerated relationships that, crucially, retained their directionality. We therefore do not believe this effect can be well explained within cognitive frameworks. A more likely explanation may thus lie in response affordances and satisficing behaviours: listed presentation makes it easier for respondents to apply uniform responses, such as dragging sliders in the same direction, reflecting perhaps fatigue or low-effort responses rather than genuine perceptual shifts in impressions. It is possible that such overarching changes in response behaviour could have resulted in the universal shift towards positive relationships between characteristics.

Importantly, taken together, these findings suggest that the listed presentation format has likely decreased data quality, which is reflected in perhaps noisier data (see inter-rater agreement and, at times, low correlations of mean ratings across presentation formats) and leads to findings that are both counter intuitive and go against existing findings in the literature. These findings call for caution when asking participants to make multiple perceptual judgements simultaneously, in response to a single stimulus presentation. However, there may be research questions and experimental procedures in which this listed presentation format may nonetheless be valid. For example, this approach may be efficient for studies that incidentally use impression ratings to facilitate deeper encoding of face identities in forensic contexts (Towler et al., 2017). Here, the impressions are only used to answer a different research question, but nevertheless, impression formation researchers may need to use caution if drawing conclusions or re-analysing these impression data specifically. Similarly, we found that the listed presentation format reduced the experiment duration by 22%. As a result, experiments in which reducing the experiment duration is paramount may benefit from the listed presentation format. Finally, mean ratings, when computed across our stimulus and participant samples, were not affected by this presentation style, such that experiments that compare means across groups could potentially use the listed presentation format (although the distributions underpinning these means seem to be affected, and it may again render the data less useful for reanalysis for other research questions).

We also want to raise the possibility that the same data that were collected here using within-subjects manipulations can also be collected by introducing between-subjects manipulations. One way of going about this would be to just ask participants to rate a single (or a small number of) characteristics, as has been done in many previous studies (Hehman et al., 2017; Lavan & Sutherland, 2024; Oh et al., 2019; Stolier, Hehman, & Freeman, 2018; Stolier et al., 2020; Willis & Todorov, 2006)). Given the substantial literature reporting replicable findings, we can assume that between-subject manipulations may also likely result in data of good quality. While there may be effects of different participant samples, the high inter-rater agreement observed for most trait ratings may limit these effects. The main shortcoming of this method is that all inferences across different characteristics are stimulus-bound, meaning we cannot draw conclusions about individual participants’ perceptions across multiple characteristics. How different within- and between-subject manipulations are for impression formation from faces, however, still needs to be formally established.

On a broader level, our current study overall highlights that task design choices can have substantial effects on the data collected. We focused here on first impressions from faces, but we see no reason why other stimuli would not be subject to the same considerations, for example, research on other subjective judgements from voices, bodies, or and beyond, given that many similarities between how first impressions are formed from these stimuli have been reported (Hu & O’Toole, 2023; Young et al., 2020; Yovel & Belin, 2013). Beyond the specific mechanisms underlying our findings, these results thus raise broader questions about the robustness of findings and theories built on the same, highly standardised experimental paradigms. If a relatively simple change in the task design for a standard ratings study can at times, have drastic effects on the data collected, we can assume that other changes to these standard paradigms, as well as using entirely different tasks (e.g. implicit measures of trait perception), may also yield different results and insights. It is therefore in the end essential – and a substantial challenge – to delineate task- or paradigm-driven patterns in our data from patterns that speak to cognitive or perceptual mechanisms.

In the longer run, it may therefore be helpful to determine how much tasks and task design choices drive (or obscure) cognitive effects. We hope that our results can serve as an example to allow researchers to more carefully weigh up different design choices for future experiments: While it is often impossible to assess, pilot, or even consider all possible options for a task design, it is possible to make some informed decisions to avoid key pitfalls in presentation format as outlined here. Not only can presentation format choices optimise data quality, but being aware of how design choices affect our data can also likely lead to more replicable results across the field and a stronger foundation for theory.
